# Altered Brain Leptin and Leptin Receptor Expression in the 5XFAD Mouse Model of Alzheimer’s Disease

**DOI:** 10.3390/ph13110401

**Published:** 2020-11-18

**Authors:** Anishchal A. Pratap, R. M. Damian Holsinger

**Affiliations:** 1 Brain and Mind Centre, Laboratory of Molecular Neuroscience and Dementia, Faculty of Medicine and Health, The University of Sydney, Sydney, NSW 2050, Australia; apra8538@uni.sydney.edu.au; 2 Discipline of Pathology, School of Medical Sciences, Faculty of Medicine and Health, The University of Sydney, Sydney, NSW 2006, Australia

**Keywords:** astrocytes, Alzheimer’s disease, glia, metabolic dysregulation, neurodegenerative disease, leptin

## Abstract

Alzheimer’s disease (AD) is a complex neurodegenerative disorder characterized by the accumulation of amyloid plaques and neurofibrillary tangles. Interestingly, individuals with metabolic syndromes share some pathologies with those diagnosed with AD including neuroinflammation, insulin resistance and cognitive deficits. Leptin, an adipocyte-derived hormone, regulates metabolism, energy expenditure and satiety via its receptor, LepR. To investigate the possible involvement of leptin in AD, we examined the distribution of leptin and LepR in the brains of the 5XFAD mouse model of AD, utilizing immunofluorescent staining in young (10–12-weeks; *n* = 6) and old (48–52-weeks; *n* = 6) transgenic (Tg) mice, together with age-matched wild-type (WT) controls for both age groups (young-WT, *n* = 6; old-WT, *n* = 6). We also used double immunofluorescent staining to examine the distribution of leptin and leptin receptor expression in astrocytes. In young 5XFAD, young-WT and old-WT mice, we observed neuronal and endothelial expression of leptin and LepR throughout the brain. However, neuronal leptin and LepR expression in the old 5XFAD brain was significantly diminished. Reduced neuronal leptin and LepR expression was accompanied by plaque loading and neuroinflammation in the AD brain. A marked increase in astrocytic leptin and LepR was also observed in old 5XFAD mice compared to younger 5XFAD mice. We postulate that astrocytes may utilize LepR signalling to mediate and drive their metabolically active state when degrading amyloid in the AD brain. Overall, these findings provide evidence of impaired leptin and LepR signalling in the AD brain, supporting clinical and epidemiological studies performed in AD patients.

## 1. Introduction

Alzheimer’s disease (AD) is a progressive neurodegenerative disease of the brain that is characterized by memory impairment and cognitive dysfunction [1,2]. Pathological hallmarks of AD are the deposition of amyloid beta (Aβ) plaques and neurofibrillary tangles [3,4]. The accumulation of these proteinaceous deposits induce synaptic dysfunction, neuroinflammation and oxidative damage, resulting in neurodegeneration [5]. Although the downstream effects of amyloid toxicity have been well documented [3,6,7], the aetiology of the disease still remains unknown.

AD has been reported as a multifactorial disease with multiple epidemiological and clinical studies reporting that patients with metabolic syndromes including type 2 diabetes mellitus (T2DM) and/or obesity are at increased risk of developing AD [8,9,10,11]. Indeed, several pathological similarities shared between metabolic syndromes and AD have been highlighted, including insulin resistance, inflammation and cognitive deficits [9,12,13,14,15]. Investigations in animal models of AD have shed further light on the association between diabetes and Alzheimer’s. A study discovered an increased amyloid precursor protein (APP) load, in addition to a decreased insulin receptor activity in the brain of diabetes-induced (via intraperitoneal administration of streptozotocin) AD-transgenic hAPP (human-APP) mice [16]. Another study investigating the behavioural responses of transgenic T2DM and AD mice, reported that both strains elicited similar learning and memory impairments, as well as increased anxiety and fear responses [12]. Additionally, a study comparing two different mouse strains of AD, the 3xTg-AD and Tg2576, reported evidence of age-related insulin signalling deficiencies in the brain, which preceded peripheral insulin resistance [17]. Finally, an investigation employing cynomolgus monkeys over 20 years of age, found that those with T2DM exhibited an increased Aβ pathology compared with age-matched controls [18]. The authors noted increased insulin resistance and impaired insulin signalling caused by Aβ cytotoxicity [18].

The satiety hormone leptin is responsible for creating a negative feedback loop that mediates feeding responses and energy metabolism [19]. Leptin is a large (16 kDa) protein that is secreted primarily by adipocytes into circulation. Due to the molecular weight of the molecule, leptin can only cross the blood–brain barrier (BBB) via selective transporters and acts primarily on arcuate nuclei in the hypothalamus to regulate satiety [20]. This is evident in leptin (ob/ob) knockout (KO) mice, as they have an increased body weight, high energy reserves in the form of hyperglycaemia and peripheral neuropathy in older mice compared to controls [21]. Leptin receptors, encoded by the db gene, have six isoforms that result from alternative mRNA splicing, including the short-forms, Lep-Ra, Lep-Rc, Lep-Rd, Lep-Re and Lep-Rf, that consist of a short (32-40 amino acid) intracellular domain and a long-form Lep-Rb (LepR) that is thought be the most bioactive due to the presence of a long, 300 amino acid, intracellular domain [22]. Binding of leptin to the receptor results in the dimerization of the LepR that triggers an increase in Janus tyrosine kinase 2 (JAK2) phosphorylation, leading to the activation of various intracellular signalling molecules. Most of these molecules play pivotal roles in signal transduction pathways such as the signal transducer and the activator of transcription 3 (STAT3), mitogen-activated protein kinase (MAPK) and phosphatidylinositol 3-kinase (PI3K) [21]. Through the utilization of these intracellular pathways, leptin contributes to the metabolic and homeostatic activity of cells.

Within the central nervous system (CNS), in addition to being densely packed in hypothalamic nuclei, there is a wide expression of leptin receptors in the hippocampus, cerebellar cortex, vagal dorsal motor nuclei and neocortex [23]. Mice with mutated leptin receptors (db/db genotype) display hyperphagia, obesity and leptin insensitivity [20]. Leptin resistance has also been observed in cases of obesity, due to reduced sensitivity of LepR to leptin, resulting in an impaired modulation of satiety [24]. Additionally, similar alterations in leptin and LepR have been reported in the cerebrospinal fluid (CSF) and brain tissue of AD subjects [25]. However, the mechanism(s) as to how leptin resistance arises in AD and neurodegenerative disorders is poorly understood.

The neuroprotective effects of leptin on Alzheimer’s have been the focus of many studies including Fewlass and colleagues [26], who reported that leptin treatment reduced Aβ load in both in-vitro and in-vivo models of AD. Additionally, Marwarha and colleagues [27] observed reduced leptin levels in the hippocampi of rabbits fed with high fat diets. The rabbit hippocampi also showed significant levels of Aβ and hyperphosphorylated tau as a result of the high fat diet, consistent with other reports [28]. Application of leptin to ex vivo rabbit hippocampal slices decreased Aβ and hyperphosphorylated tau load [27]. These studies indicate that leptin plays a protective role against Aβ in the brain. Most studies have focused on the response of cells, typically neurons, to the effect of leptin in the CNS. We therefore, focused our study on the expression pattern of leptin and its receptor (LepR) in the 5XFAD mouse model of AD, examining both early and late stages of the disease to determine the role of leptin and LepR expression during AD progression.

## 2. Results

### 2.1. Leptin and LepR Expression in the Cerebral Cortex of Young- and Old-5XFAD (Tg) and Wild-Type (WT) Mice

**Figure 1:**
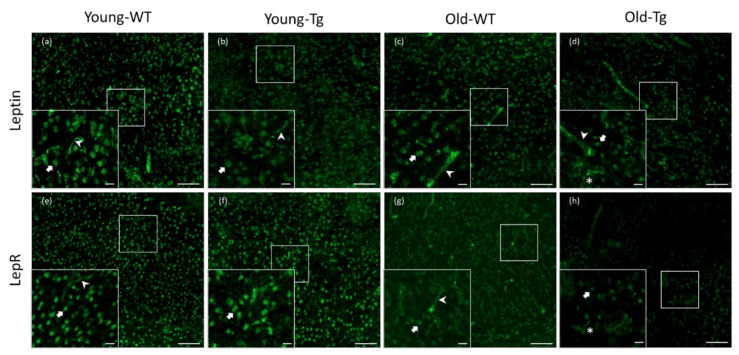
Expression of leptin and leptin receptor (LepR) in cerebral cortex of young (10–12 week) and old (48–52 week) wild-type (WT) and 5XFAD (Tg) mice. Leptin was detected in neurons and endothelial cells in (**a**) young-WT, (**b**) young-Tg and (**c**) old-WT mice. Leptin was detected in neurons and glia in (**d**) old-Tg mice. LepR expression was also detected in neurons and endothelial cells of (**e**) young-WT, (**f**) young-Tg and (**g**) old-WT mice. Moreover, LepR was observed to be expressed by neurons as well glia in (**h**) old-Tg mice. Block arrows—neurons; pointed arrows—endothelial cells; *—glia. Scale bar for large figures, 100 μm; smaller figures in bottom left, 20 μm.

Visualization of stained sections revealed neuronal and endothelial leptin and LepR expression in the WT and 5XFAD mice (Figure 1). Qualitatively, we observed a difference in neuronal expression of both leptin and LepR in the cerebral cortex between young (young-Tg, Figure 1b and Figure 1f, respectively) and old 5XFAD mice (old-Tg, Figure 1d and Figure 1f, respectively). Moreover, we observed glial expression of leptin (Figure 1d) and LepR (Figure 1h) in the old-Tg (48–52-week-old) mice, whereas old-WT mice only showed reduced neuronal leptin (Figure 1c) and LepR (Figure 1g) expression. Overall, we observed a qualitative decrease in expression of both leptin and LepR in the old-WT and old-Tg mice compared with the younger WT and Tg mice. This indicates that leptin and LepR expression may be downregulated with ageing.

### 2.2. Quantitative Analysis of Cortical Neuronal Leptin and LepR Expression

**Figure 2:**
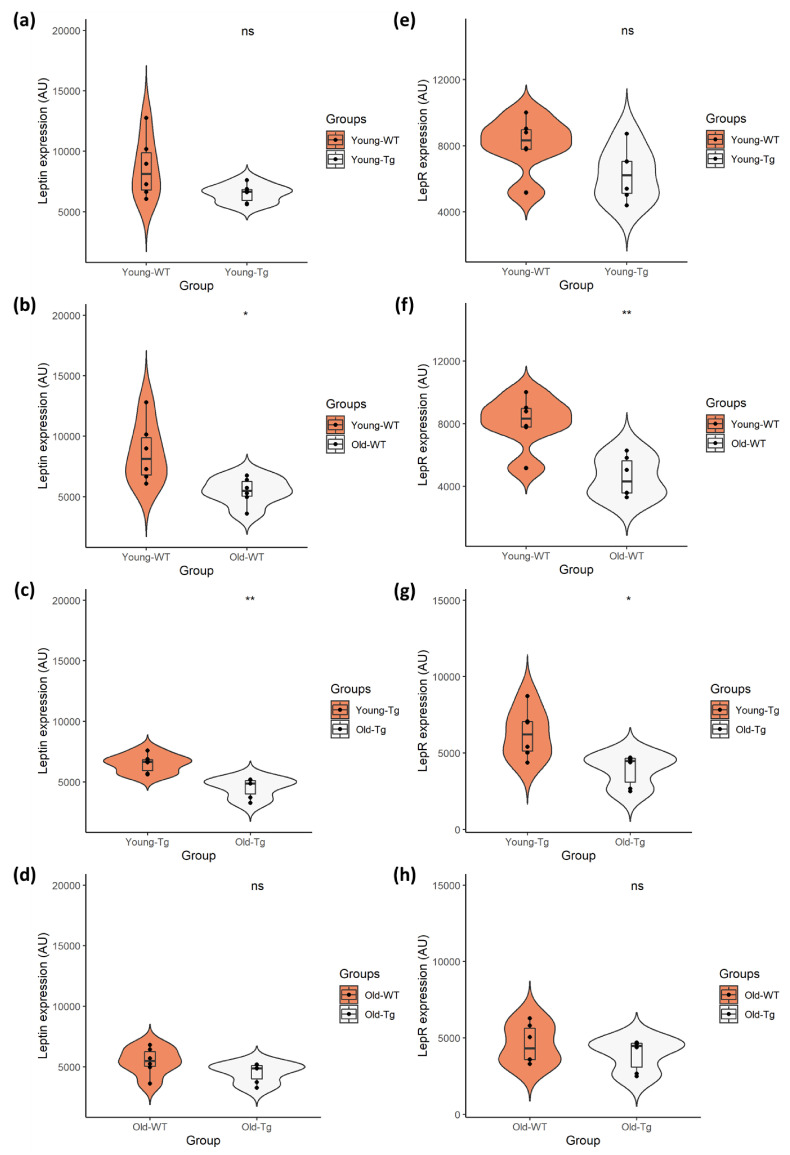
Quantitative analysis of neuronal leptin and LepR expression in young (10–12 week) and old (48–52 week) 5XFAD (Tg) mice and wild-type (WT) controls. Independent t-test analysis of (**a**) neuronal leptin and (**e**) LepR expression between the young-WT and young-Tg revealed no significant differences. However, there was significant difference in expression of (**b**) leptin and (**f**) LepR between the young-WT and old-WT groups. This was also observed with the (**c**) leptin and (**g**) LepR expression amongst young-Tg and old-Tg mice. However, there was no significant difference in (**d**) leptin or (**h**) LepR expression between the old-WT and old-Tg mice. Data presented as violin plots using an independent t-test, with statistical significance indicated as * *p* < 0.05 and ** *p* < 0.01 between groups.

Quantitative analyses revealed no difference (*t*(10) = 5.886, *p* < 0.09, Cohen’s *D* = 1.14) in expression of neuronal leptin (Figure 2a) between the young-WT (*M* = 8638.60, *SD* = 2526.71) and young-Tg (*M* = 6514.98, *SD* = 754.88) groups. There was also no difference (*t*(10) = 9.993, *p* < 0.081, Cohen’s *D* = 1.21) in neuronal LepR expression (Figure 2e) between young-WT (*M* = 8103.67, *SD* = 1659.65) and young-Tg (*M* = 6266.98, *SD* = 1615.97) mice. Although our data presents a marginal reduction in leptin and LepR expression between the WT and 5XFAD mice, there was no significant difference in leptin expression (Figure 2d) (*t*(10) = 9.016, *p* < 0.135, Cohen’s *D* = 0.95) between the old-WT (*M* = 5458.91, *SD* = 1144.02) and old-Tg (*M* = 4517.60, *SD* = 811.70) mice, nor was there a significant difference in LepR expression (Figure 2h) (*t*(10) = 9.581, *p* < 0.334, Cohen’s *D* = 0.59) amongst the old-WT (*M* = 4601.83, *SD* = 1287.44) and Tg (*M* = 3913.46, *SD* = 1041.10) mice.

Conversely, a significant decrease (*t*(10) = 6.967, *p* < 0.02, Cohen’s *D* = 1.62) in neuronal leptin (Figure 2b) was observed between young-WT (*M* = 8638.60, *SD* = 2526.71) and old-WT (*M* = 5458.91, *SD* = 1144.02) mice. This decrease (*t*(10) = 9.418, *p* < 0.002, Cohen’s *D* = 2.36) was also evident in neuronal LepR expression (Figure 2f) between young-WT (*M* = 8103.67, *SD* = 1659.65) and old-WT (*M* = 4601.83, *SD* = 1287.44) groups. Similarly, a significant decrease (*t*(10) = 9.948, *p* < 0.001, Cohen’s *D* = 2.55) in neuronal leptin (Figure 2c) was observed between the young-Tg (*M* = 6514.98, *SD* = 754.88) and old-Tg (*M* = 4517.60, *SD* = 811.70) mice. A significant reduction (*t*(10) = 8.541, *p* < 0.016, Cohen’s *D* = 1.73) in LepR expression (Figure 2g) was also detected amongst the young-Tg (*M* = 6266.98, *SD* = 1615.97) and old-Tg (*M* = 3913.46, *SD* = 1041.10) groups. Thus, the reduction of brain leptin and LepR expression occurs as the mice age, with no significant effect from genotypes.

### 2.3. Amyloid Plaque Loading and Astrocytic Expression of Leptin and LepR in Hippocampi of Aged-5XFAD Mice

**Figure 3:**
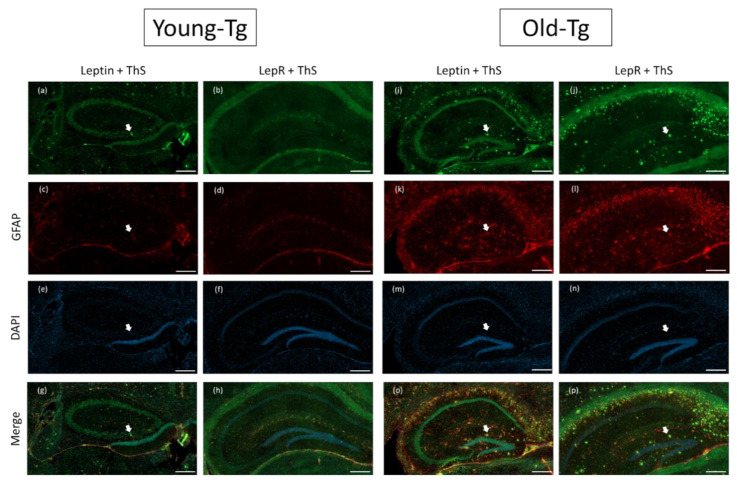
Leptin and LepR with Thioflavin-S and glial fibrillary acidic protein (GFAP) staining on the hippocampi of young (10–12 week) and old (48–52 week) 5XFAD (Tg) mice. Amyloid plaques stained by thioflavin-S (ThS) are indicated by white arrows. Plaques were more prevalent in the hippocampi of old-Tg (**i**,**j**,**o**,**p**) compared to young-Tg mice (**a**,**b**,**g**,**h**). Leptin and LepR was detected in the hippocampus of young-Tg (**a** and **b**, respectively) and to a lesser extent in old-Tg mice (**i** and **j**, respectively). Both young- (**c**,**d**) and old-Tg (**k**,**l**) mice also had astrogliosis identified by GFAP staining. Furthermore, astrocytic expression of leptin (**o**) and LepR (**p**) with GFAP was observed in the hippocampi of old-Tg mice compared to young-Tg mice (**g** and **h**, respectively). Young- (**e**,**f**) and old-Tg (**m**,**n**) sections were counterstained with 4′,6-diamidino-2-phenylindole (DAPI). Scale bar = 200 μm.

In our immunofluorescent experiments, we utilized thioflavin-S (ThS) to stain for amyloid plaques. Along with reports from others [29,30], we observed deposition of amyloid plaques in the hippocampi (Figure 3) of old-Tg (48–52 week) mice and young-Tg (10–12 week) mice. Amyloid deposition was observed to be greater in old-Tg mice (Figure 3i-p) compared to young-Tg mice (Figure 3a–h). We had previously reported that 48–52-week-old 5XFAD mice had an increased astrocytic expression of glial fibrillary acidic protein (GFAP) [31]. Similarly, we observed increased astrocytic GFAP expression in the old-Tg mice (Figure 3f–k) compared to young-Tg mice (Figure 3c, d). We postulate this to be a result of an increased amyloid burden. Decreased neuronal expression of leptin (Figure 3i) and LepR in the hippocampi (Figure 3j) of old-Tg mice compared to younger animals (Figure 3a and Figure 3b, respectively) was observed. Conversely, the old-Tg mice had an increased astrocytic expression of leptin (Figure 3o) and LepR (Figure 3p) compared to young-Tg animals (Figure 3g and Figure 3h, respectively). These results indicate a shift in leptin and LepR expression in the brain as the 5XFAD mice and their WT littermates aged. Furthermore, colocalization of leptin and GFAP in astrocytes provides evidence of leptin as a modulator of astrocytic LepR expression in the brain.

### 2.4. Astrocytic Expression of Leptin and LepR in Aged-5XFAD Mice

**Figure 4:**
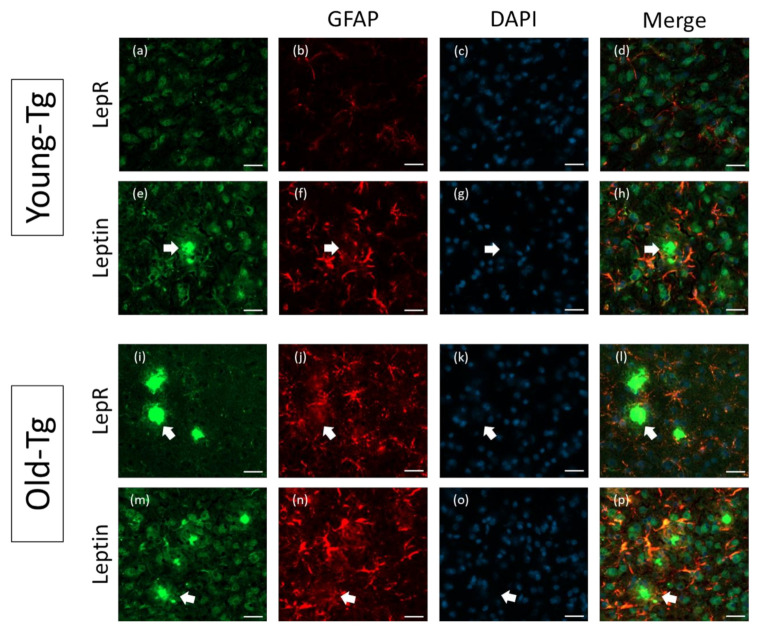
Astrocytic expression of leptin and LepR in the cerebral cortex of young (10–12 week) and old- (48–52 week) 5XFAD (Tg) mice. Thioflavin-*S*-stained amyloid plaques (arrows) are present in the cerebral cortex of old-Tg and to a lesser extent in young-Tg mice. LepR and leptin was detected in neurons and astrocytes in the cerebral cortex of young- (**a** and **e**, respectively) and old-Tg (**i** and **m**, respectively) mice. Panels (**b**,**f**,**j**,**n**) depict astrocytes labelled with GFAP. Panels (**c**,**g**,**k**,**o**) depict nuclei staining with DAPI. Colocalization of LepR with GFAP (**l**) and leptin with GFAP (**p**) labelled astrocytes of old-Tg mice was observed. Co-expression of LepR (**d**) and leptin (**h**) with GFAP was also observed to a lesser extent in young-Tg mice. Scale bar = 20 μm.

Further analysis of leptin and LepR expression in the Tg mouse brain was performed on the cerebral cortex of young- (10–12 week) and old-Tg (48–52 week) mice. Qualitatively, LepR (Figure 4a) and leptin (Figure 4e) immunoreactive cells were observed surrounding amyloid plaques in the cerebral cortex of old-Tg mice. Colocalization of both LepR (Figure 4d) and leptin with GFAP (Figure 4h) was also apparent in the cortex. As mentioned above, a high expression of GFAP (Figure 4b,f) in the old-Tg mice was observed compared to the young cohort (Figure 4j,n). Additionally, the GFAP-stained astrocytic processes were enlarged and more numerous in the old-Tg mouse compared to the young-Tg mice. This coincides with the inflamed state of the late-stage AD brain, and the role astrocytes play in amyloid plaque clearance [32]. Astrocytes were observed to surround amyloid aggregates in both young- and old-Tg mice (shown by arrows in Figure 4). Furthermore, while astrocytes proximal to the amyloid deposits expressed leptin (Figure 4p) and LepR (Figure 4l), astrocytes distal to plaques expressed GFAP but were devoid of leptin or LepR immunoreactivity in both Tg age groups (Figure 4h,l,p), probably reflective of the metabolically “active” nature of astrocytes surrounding the plaques.

**Figure 5:**
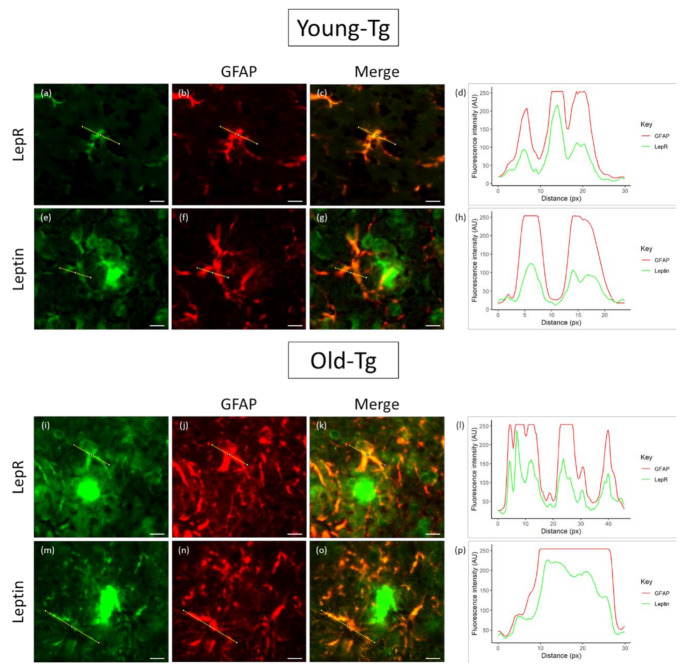
Colocalization of leptin and LepR with glial fibrillary acidic protein (GFAP) in astrocytes of young (10–12 week) and old (48–52 week) 5XFAD (Tg) mice. Thioflavin-*S* was used to stain for amyloid plaques. LepR and leptin were detected in astrocytes of both young- (**a** and **e**, respectively) and old-Tg mice (**l** and **m**, respectively). Astrocytes in old-Tg mice had high expression of GFAP (**j**,**n**) compared to young mice (**b**,**f**). A high degree of colocalization of GFAP with LepR and leptin in astrocytes was observed in old (**k** and **o**, respectively) compared to young-Tg (**c** and **g**, respectively) mice. The line expression profile of GFAP and LepR (**d**) in the astrocytes of young-Tg mice presented similar peaks and troughs along the same relative distance (yellow line). The expression profile of GFAP and leptin (**h**) shows low levels of leptin (yellow line) in astrocytes of young-Tg mice. Expression profile of GFAP and LepR (**l**) in the astrocytes of old-Tg mice had similar intensities to young-Tg mice. The intensity profile of GFAP and leptin (**p**) showed increased expression of leptin in the astrocytes of old-Tg mice. Scale bar = 10μ m.

To explore the colocalization of leptin and LepR, we analysed their relative intensities by the separation of red and green color channels and determined the plot profile of signal intensity along a specified distance (across the astrocyte cell body and processes) (Figure 5). In young-Tg mice, astrocytes proximal to plaques expressed both LepR (Figure 5a) and leptin (Figure 5e) along with GFAP (Figure 5b,f). However, the expression profiles of LepR and GFAP revealed that the relative intensities of these two proteins (Figure 5d) had similar peaks and troughs along the same relative distance (yellow line in Figure 5a–c). However, although the expression profile of GFAP was high, the fluorescence intensity of leptin in astrocytes was relatively low (Figure 5h). When compared to old-Tg mice, the qualitative intensity of LepR (Figure 5i) and leptin (Figure 5m) is relatively higher than that of the young-Tg mice. Moreover, the line profile for LepR and GFAP (Figure 5l) indicates similar levels of expression across the astrocyte. Additionally, the intensity of leptin expression in the astrocyte mirrors that of GFAP (Figure 5p). These findings may indicate that astrocytes utilizes leptin to fuel its metabolic role (via binding to LepR).

## 3. Discussion

A growing body of evidence suggests that individuals with metabolic syndromes such as T2DM, obesity and hypercholesterolemia predispose them to AD [25,33,34,35,36,37,38]. Additionally, many metabolic-associated conditions and neurodegenerative diseases, including AD, display signs of chronic inflammation, which induces an activated glial response [39,40,41,42,43,44]. Leptin is a satiety hormone that mediates feeding responses and energy metabolism but has also been found to be neuroprotective against toxic Aβ in-vitro and prevents amyloid plaque build-up in mouse models of AD [26,45]. However, studies regarding the endogenous expression of leptin and its receptor (LepR) in mouse models of AD have been limited. Therefore, to provide a better understanding of impaired metabolism in the AD brain, we sought to identify the expression and distribution of leptin and LepR in the brain of young (10–12 week) and old (48–52 week) 5XFAD mice.

We identified widespread expression of leptin and LepR in the WT and Tg mouse brain (Figure 1). The expression of leptin and LepR in WT mice were consistent with previous reports [46,47]. Our study provides the first evidence of altered leptin and LepR expression in the aged 5XFAD mouse brain. We report that both leptin and LepR are expressed in the neurons of young and old WT and Tg mice. Additionally, endothelial expression of leptin and LepR was found in young-WT, young-Tg and old-WT, but not in old-Tg mice. Leptin is known to be transported into the brain through selective transporters (LepRa) expressed by pericytes, endothelia and cells of the choroid plexus [48]. It has been reported that tight junction proteins including claudin-5 and CD31, components of the BBB, were significantly decreased in 5-month-old 5XFAD mice [49]. Therefore, it is plausible that leptin transport into the aged 5XFAD brain via LepRa may be impaired by a disruption to the BBB. Alternatively, one could assume that leptin could “leak” through the BBB and permeate the AD brain. However, qualitatively, we found lower levels of leptin in neurons of old-Tg mice compared to young-Tg animals (Figure 2).

In our quantitative assessment of neuronal expression, we found no significant differences in leptin or LepR expression between young-WT and young-Tg animals. Although not statistically significant, we did note reduced neuronal leptin and LepR expression in old-Tg mice compared to age-matched WT littermates. However, there were significant reductions in neuronal leptin and LepR expression between the young and old mice of both genotypes. This provides further evidence of leptin impairment in the ageing brain, as the ability to use leptin for metabolism and energy regulation in the brain is altered by a reduction in neuronal LepR. A similar observation was reported by King and colleagues who found an age-related decline of leptin expression in APP/PS1 and WT mice [22]. They also reported an increase in LepR levels in 9- and 18-month-old APP/PS1 mice compared to controls. We however observed an age-related decline in both leptin and LepR expression in 5XFAD and WT mice. This discrepancy may be due to the different techniques employed in the two studies. We report specific decreases in the expression of leptin and LepR in neurons in the cerebral cortex, whereas the technique of immunoblotting utilized by King et al. employed total tissue homogenates (neurons and glia). Our study provides details on the expression pattern of leptin and LepR in the 5XFAD brain and provides an insight into how leptin may be involved in AD pathology.

In addition to neurons, leptin receptor expression has been reported in glial cells including astrocytes, microglia and oligodendrocytes in the CNS [50]. We too observed leptin and LepR expression in activated astrocytes in both young and old Tg mice. We propose that astrocytic expression of these proteins reflect the nutritional demands placed upon glial cells during all phases of AD development, whereby they clear Aβ and consequent plaques formed in the brain [51,52]. In support, we observed GFAP-positive astrocytes surrounding amyloid plaques in both young- and old-Tg mice (Figure 4 and Figure 5). Additionally, we noted that leptin and LepR expression in the astrocytes of old-Tg mice were markedly higher than those of young-Tg mice. Furthermore, co-expression of GFAP with leptin and LepR was exhibited by astrocytes proximal to plaques, whereas astrocytes distal to plaques displayed low colocalization of GFAP with leptin or LepR. We propose this as evidence that astrocytes surrounding plaques are metabolically active and utilize sources of energy such as leptin-LepR binding to fuel their active state. In a recent study exploring LepR expression, Cecon and colleagues reported that Aβ bound, with high affinity, to the extracellular domain of LepR expressed on neurons [53]. This allosteric interaction may inhibit the function of LepR and consequently prevent leptin from binding. It is also plausible that the binding of Aβ to LepR on neurons causes a downregulation of the receptor. Furthermore, to test the effects of Aβ on astrocytes, Allaman et al. exposed cultured astrocytes to Aβ_25–35_ and found increased glucose metabolism in the cells [54]. Addition of Aβ_25–35_ peptides to co-cultures of neurons and astrocytes resulted in impaired neuronal viability. The effect on neurons was abrogated by pre-treatment with PI3K inhibitors, leading the authors to conclude that the deleterious effects were mediated via PI3K signalling [54]. Additionally, leptin treatment was found to inhibit Aβ fibrillogenesis, also via the PI3K pathway [55]. Our results demonstrate a switch in LepR expression from neurons to astrocytes as the disease progresses in 5XFAD mice. These results may in fact be driven by amyloid load in the mouse brain. It is plausible that Aβ blocks and downregulates the LepR on neurons, resulting in a switch in LepR expression to astrocytes that in turn need to be fuelled to perform their task of degrading Aβ. It would be important to determine whether the extracellular binding pocket of the LepR expressed by astrocytes is identical to that expressed on neurons and why Aβ does not bind to LepRs expressed on astrocytes.

To determine the role of LepR in astrocytes, Naranjo et al. selectively downregulated LepR expression in GFAP-positive cells in the hippocampi of mice and found that reduction in LepR reduced basal synaptic transmission [56]. In addition, they also demonstrated that LepRs were involved in the maintenance of glutamate homeostasis and neurotransmission within the hippocampus, highlighting an involvement of LepRs in crosstalk between neurons and astrocytes in the hippocampus [35]. This may also suggest that astrocytes could participate in hippocampal-dependent memory processes via LepR signalling. In agreement with Naranjo et al., leptin was found to induce adult hippocampal neurogenesis in adult rat progenitor cells and C57BL/6J mice [57]. Additionally, adult-onset obese agouti viable yellow (A^vy^) mice were reported the have increased astrocytic LepR in brain regions including the arcuate nucleus and dorsomedial hypothalamus [58]. Pan et al. used fluorocitrate to inhibit astrocytic activity in A^vy^ mice to determine LepR activity [59]. They discovered that LepR expression was decreased when astrocytic activity was inhibited, but also noted an increased distribution of neuronal LepR [59]. We provide further evidence of the dysregulation in astrocytic leptin and consequent LepR expression in the 5XFAD mice. Line profiles across astrocytes revealed an increase in leptin expression in old-Tg mice compared to the young group. Intriguingly, there were no differences in the overall level of expression of LepR between the Tg age groups. Therefore, it is possible that a change in leptin utilization and LepR expression occurs in neurons and astrocytes in the AD brain where astrocytes utilize leptin by increasing LepR expression to fuel its metabolic activity in degrading amyloid. This may also contribute to the disrupted neuronal metabolism, which together with Aβ-mediated damage would exacerbate neurodegeneration [60,61]. We have previously provided evidence demonstrating altered adiponectin signalling in old 5XFAD mice [44], which together with our current results provide critical evidence of metabolic dysfunction in the AD brain. Further studies focusing on the role of leptin and LepR signalling in the human AD brain as well as in other neuroinflammatory diseases involving reactive astrocytes is also warranted.

## 4. Materials and Methods

### 4.1. Chemicals and Reagents

Primary antibodies used for immunofluorescent staining included; Rabbit Anti-Leptin (Cat# PA1-052, RRID: AB_325787, ThermoFisher Scientific, Melbourne, VIC, Australia), Goat Anti-Leptin Receptor (Cat# L9536, RRID: AB_260442, Sigma-Aldrich, North Ryde, NSW, Australia) and Mouse Anti-GFAP (glial fibrillary acidic protein, Abcam, Melbourne, VIC, Australia) (Cat# ab10062, RRID: AB_296804, Abcam, Melbourne, VIC, Australia). Secondary antibodies included; Goat Anti-Rabbit IgG H&L Alexa Fluor^®^ 488 (Cat# ab150077, RRID: AB_2630356, Abcam, Melbourne, VIC, Australia), Goat Anti-Mouse Alexa Fluor^®^ 594 (Cat# ab150116, RRID: AB_2650601, Abcam, Melbourne, VIC, Australia) and Donkey Anti-Goat IgG H&L Alexa Fluor^®^ 488 (Cat# ab150129, RRID: ab_2687506, Abcam, Melbourne, VIC, Australia). DAPI (4′,6-diamidino-2-phenylindole) (Cat# D9542, Sigma-Aldrich, North Ryde, NSW, Australia) was used to stain nuclei. Thioflavin-*S* (Cat# T1892, Sigma-Aldrich, North Ryde, NSW, Australia) was used in staining and visualizing β-amyloid plaques.

### 4.2. Animals and Tissue Collection

The 5XFAD transgenic (Tg) mouse model of AD was used for this study due to their robust deposition of amyloid plaques and exhibition of neurodegeneration and gliosis as early as 2 months of age [30,62]. The Tg mice are heterozygous for five familial AD mutations including, amyloid precursor protein (APP) (APP KM670/671NL + I716V + V7171) and presenilin 1 (PSEN1) (PSEN1 M146L + L286V) [30]. Male wildtype (WT) C57BL6 mice were bred with female Tg mice to produce heterozygous offspring. Mice were housed with littermates, exposed to 12 h light–dark cycles and had free access to food and water. The age groups studied consisted of 10–12-week-old young-WT (*n* = 6) and Tg (*n* = 6) and 48–52-week-old aged-WT (*n* = 6) and Tg (*n* = 6) mice. Additionally, the allocation of both male and female mice to each age group was randomised. All animals were maintained at The University of Sydney Laboratory Animal Service facilities and were bred under protocol AEC2016/964 (approved by The University of Sydney Animal Ethics Committee on 18 March 2016). All procedures were performed in accordance with University and governmental guidelines.

Animals were deeply anaesthetised with pentobarbital before decapitation. Following decapitation, the brain was separated into two parts—the cerebral cortex and cerebellum. Each part of the brain was immersed in Shandon™ Cryomatrix™ (Cat. No. 6769006, ThermoFisher Scientific, Melbourne, VIC, Australia) medium, snap frozen in the vapour phase of liquid nitrogen and stored at −80 °C until analysis.

### 4.3. Immunofluorescence

A Leica CM1950 cryostat was used to section cortical tissues at 16µm. The sections were mounted and stored on glass slides at −20 °C. Glass slides with frozen sections were thawed at room temperature (RT) briefly before fixation in ice cold methanol (100%). Post-fixation, the sections were rinsed in a phosphate buffered saline (PBS) and washed twice, followed by a 30 min incubation at RT in 1% goat serum (Cat. No. ab7481, RRID: AB_2716553, Abcam, Melbourne, VIC, Australia) in PBS. Following a brief wash in PBS, the sections were incubated with the primary antibodies; Rabbit Anti-Leptin (1:150) or Goat Anti-LepR (1:150) and Mouse Anti-GFAP (glial fibrillary acidic protein) (1:600) overnight at 4 °C. Following removal of the primary antibodies and three subsequent PBS washes, the sections were incubated with the following secondary antibodies; Goat Anti-Rabbit IgG H&L Alexa Fluor^®^ 488 (1:300) or Donkey Anti-Goat IgG H&L Alexa Fluor^®^ 488 (1:300) and Goat Anti-Mouse Alexa Fluor^®^ 594 (Cat No. ab150116, AB_2650601, AB_2650601) (1:300) for 1 h at RT in the dark. Sections were washed with PBS and counter-stained with DAPI (Cat No. D9542, Sigma Aldrich) briefly before coverslipping with DPX mountant (Cat No. 06522, Sigma-Aldrich, Sigma-Aldrich, North Ryde, NSW, Australia). The slides were stored at 4 °C prior to image analysis. Sections were imaged using a Zeiss Axio Scan.Z1 slide scanner (Carl Zeiss, Oberkochen, Germany).

### 4.4. Thioflavin-S Staining

The procedure for amyloid plaque-labelling with thioflavin-S (ThS) has been reported by our group previously [31]. Briefly, 0.015% ThS (in 50% ethanol *w*/*v*) was added to each section following the final PBS wash in the immunofluorescent staining above. Following 10 min of incubation in the dark, excess stain was washed off the sections through dehydrating washes of 80% ethanol, 80% ethanol and 95% ethanol. Sections were then rehydrated with double-distilled water three times prior to coverslipping. Tissue sections were also imaged using the Zeiss Axio Scan.Z1 slide scanner (Carl Zeiss, Oberkochen, Germany).

### 4.5. Quantification and Statistical Analysis

#### 4.5.1. Image Analysis

Staining quantification: Protein quantification analyses was performed using ImageJ version 1.8.0_172 (ImageJ, National Institutes of Health, Bethesda, MD, USA) as previously described [31]. Briefly, to determine neuronal protein levels, ten random neuronal cells per brain section were outlined with the freehand tracer tool in ImageJ. Fluorescence intensities of the neurons were subtracted from the background intensities of areas adjacent to the cells and were averaged.

Line profiles: Colocalization of two proteins was also measured using ImageJ. Briefly, a magnified image of an astrocyte was split into its respective colour channels (i.e., red and green). A line was drawn crossing the astrocyte (including process and body) on one image, with the line also crossing the same region on the other colour channel using the ROI (region of interest) manager. The plot profiles for each channel were obtained, merged, and presented using R version 3.6.3.

#### 4.5.2. Statistical Analysis

An independent sample t-test was utilized to determine the significance between the young-WT and young-Tg (10–12-week-old), young-WT and old-WT (48–52-week-old), young-Tg and old-Tg and between old-WT and old-Tg age groups, with significance indicated at * *p* < 0.05 and ** *p* < 0.01. Statistical analyses were conducted using R version 3.6.3 and SPSS version 25 (IBM).

## 5. Conclusions

Our study provides further evidence of an impaired metabolic environment in the 5XFAD mouse brain. We report significantly decreased neuronal expression of leptin and its receptor (LepR), but an overexpression of these two proteins in activated astrocytes of aged 5XFAD mice. Our results demonstrate that progression of Alzheimer’s pathology triggers astrocytes to robustly upregulate LepR expression. This may act as a fuelling mechanism in astrocytes to fulfil their job of degrading amyloid plaques and protecting the brain from a myriad of toxic by-products from neuroinflammation, but also indirectly leading to energy deficits in neurons in AD.
